# Site-Directed Genome Knockout in Chicken Cell Line and Embryos Can Use CRISPR/Cas Gene Editing Technology

**DOI:** 10.1534/g3.116.028803

**Published:** 2016-03-25

**Authors:** Qisheng Zuo, Yinjie Wang, Shaoze Cheng, Chao Lian, Beibei Tang, Fei Wang, Zhenyu Lu, Yanqing Ji, Ruifeng Zhao, Wenhui Zhang, Kai Jin, Jiuzhou Song, Yani Zhang, Bichun Li

**Affiliations:** * Key Laboratory of Animal Breeding Reproduction and Molecular Design for Jiangsu Province, College of Animal Science and Technology, Yangzhou University, Yangzhou 225009, China; † Animal and Avian Sciences, University of Maryland, College Park, Maryland 20741

**Keywords:** CRISPR/Cas, chicken, gene knockout

## Abstract

The present study established an efficient genome editing approach for the construction of stable transgenic cell lines of the domestic chicken (*Gallus gallus domesticus*). Our objectives were to facilitate the breeding of high-yield, high-quality chicken strains, and to investigate gene function in chicken stem cells. Three guide RNA (gRNAs) were designed to knockout the *C2EIP* gene, and knockout efficiency was evaluated in DF-1 chicken fibroblasts and chicken ESCs using the luciferase single-strand annealing (SSA) recombination assay, T7 endonuclease I (T7EI) assay, and TA clone sequencing. In addition, the polyethylenimine-encapsulated Cas9/gRNA plasmid was injected into fresh fertilized eggs. At 4.5 d later, frozen sections of the embryos were prepared, and knockout efficiency was evaluated by the T7EI assay. SSA assay results showed that luciferase activity of the vector expressing gRNA-3 was double that of the control. Results of the T7EI assay and TA clone sequencing indicated that Cas9/gRNA vector-mediated gene knockdown efficiency was approximately 27% in both DF-1 cells and ESCs. The CRISPR/Cas9 vector was also expressed in chicken embryos, resulting in gene knockdown in three of the 20 embryos (gene knockdown efficiency 15%). Taken together, our results indicate that the CRISPR/Cas9 system can mediate stable gene knockdown at the cell and embryo levels in domestic chickens.

Gene editing is an important method for studying gene function (Zhang *et al.* 2014; Jin *et al.* 2014; Huang *et al.* 2014; Fu *et al.* 2012), and gene knockout and knock-in technologies have been well established in mammals. For example, Geurts *et al.* (2009) knocked out an exogenous gene encoding green fluorescent protein (GFP), and two endogenous genes (*IgM* and *Rab38*) in rat embryos using zinc finger nuclease (ZFN) technology, and demonstrated functional loss of the genes in transgenic rats. H. Wang *et al.* (2013) generated a mouse model with disruptions and insertions in the *Sry* and *Uty* genes using transcription activator-linked nuclease (TALEN) technology, demonstrating the usefulness of this tool to study the function of Y chromosome genes. Although ZFN and TALEN are widely used techniques, their application is limited because of the difficulty of construct design and high off-target rates (Gaj *et al.* 2013). In contrast, the clustered regularly interspaced short palindromic repeats (CRISPR)/CRISPR-associated (Cas) system has a high success rate (80%), and simpler construct design (Hwang *et al.* 2013).

CRISPR/Cas9 was initially used to modify the genomes of mammalian cells such as monkey embryonic stem cells (ESCs). Recently, this technology was used in human cells to develop a gene therapy approach for Fanconi anemia. CRISPR/Cas9 technology is gradually being used at an individual level. For example, Niu *et al.* (2014) injected guide RNA (gRNA) and Cas9 RNA into monkey oocytes to modify three target genes, and Hwang *et al.* (2013) modified the *drd3* and *gsk3b* genes in zebrafish embryos to obtain a two-locus mutant.

Cong and Zhang (2015) have modified the CRISPR system to edit any gene in living cells. This system can also be used to identify genes involved in specific diseases, such as those related to antitumor drug resistance in melanoma cells. The CRISPR system can also achieve gene knock-in (Shalem *et al.* 2014). Using the CRISPR system, Lijuan *et al.* (2015) successfully cured a genetic disease causing cataracts in mice, and Schwank *et al.* (2013) corrected a gene deficiency responsible for cystic fibrosis in human stem cells.

Although CRISPR/Cas9-mediated gene editing has been widely used in humans (Mali *et al.* 2013), mice (H.Y. Wang *et al.* 2013), zebrafish (Chang *et al.* 2013), and plants (Bortesi and Fischer 2015), this method has not yet been used in poultry. Since 1997, our laboratory has studied the regulation of embryo development and differentiation of ESCs to male germ cell lines in domestic chickens (*Gallus gallus domesticus*). We sequenced the male germ cell transcriptome throughout the developmental stages, and identified genes and signaling pathways involved in ontogenesis of male germ cells (Zhang *et al.* 2015). To characterize the functions of these genes and signaling pathways, it is necessary to establish an effective *in vitro* gene editing system. Here, we describe a novel CRISPR/Cas9-mediated gene knockout approach for the rapid construction of stable transgenic cell lines of the domestic chicken. The efficiency of gene knockout was confirmed in DF-1 chicken fibroblasts, chicken ESCs, and chicken embryos using the target gene *C2EIP*, which is especially expressed in primordial germ cells. Our objective was to facilitate the characterization of the differentiation of ESCs to male germ cells and the breeding of novel high-yield and high-quality chicken strains.

## Materials and Methods

### Ethics statement

The procedures involving animals and their care conformed to the U.S. National Institutes of Health Guidelines (NIH Pub. No. 85-23, revised 1996). The experiments were approved by the Ethics Committee of Yangzhou University for Laboratory and Experimental Animals. The Suqin yellow chickens used in this study were provided by the Institute of Poultry Science, Chinese Academy of Agriculture Sciences. The experimental procedures were approved by the Institutional Animal Care and Use Committee of Yangzhou University.

### Gene cloning and gRNA design

To clone the target gene (*C2EIP*), we designed the following primers based on the mRNA sequence obtained from the NCBI database (ID: XM_001233327.1): forward, GAGGCTATCAAATGGCAG; reverse, TCACCCAATGAAAATAAAT. We identified the coding sequence region based on the protospacer adjacent motif (NGG or GGN), using the initial 19–21 bp for gRNA design. To avoid off-target effects, the entire genome was searched for potential off-target sites. The gRNA sequences are shown in Table 1.

**Table 1:** CRISPR gRNA sequences

	gRNA sequence	PAM
gRNA-1(g1)	CTTTTCTGTGCCATTCTCCA	AGG
gRNA-2(g2)	AGCACAGAGGAGTTCCTCTG	AGG
gRNA-3(g3)	ACACGCTGCTTTCATAGTCCAA	TGG

gRNA, guide RNA; PAM, protospacer adjacent motif.

### Construction of CRISPR/Cas9 vector

**Figure 1:**
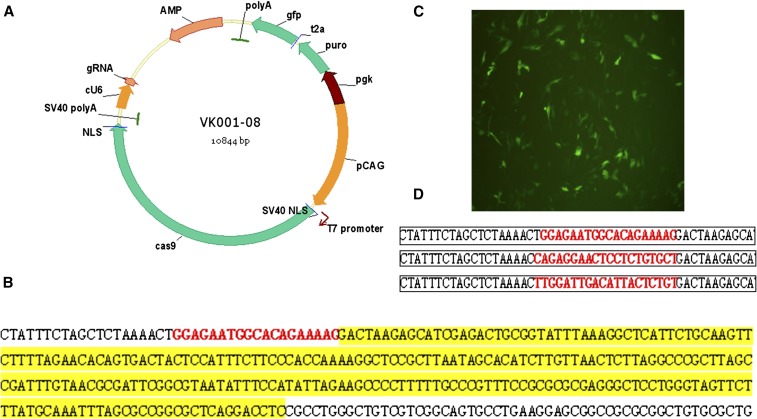
Construction of the CRISPR/Cas9 vector. (A) Schematic diagram of the CRISPR/Cas9 vector. (B) Sequencing results after an avian-derived U6 promoter was inserted into the vector. (C) GFP expression after DF-1 cells were transfected with VK001-08. (D) Sequencing results after gRNA was inserted into the vector.

The target site sequence of gRNA was inserted into the CRISPR/Cas9 vector (Figure 1A), which expresses Cas9 protein and gRNA simultaneously using the T7 and avian-derived U6 promoters, respectively. To facilitate cell screening, this plasmid expresses the puromycin resistance gene and GFP.

### SSA assay, T7 endonuclease I (T7EI) assay, and TA clone sequencing

For construction of the single-strand annealing (SSA) luciferase reporter, we inserted a terminator sequence followed by the gRNA target sequence into a plasmid encoding the luciferase gene. The SSA luciferase reporter was cotransfected with the CRISPR/gRNA vector and a Renilla luciferase reporter (internal control). An empty plasmid was used as a negative control.

At 48 hr after transfection, GFP-positive cells were selected by flow cytometry from the second-generation ESCs with high cleavage activity. The T7EI assay was performed using genomic DNA from GFP-positive cells. Polymerase chain reaction (PCR) amplification of the region containing the target site was carried out using the following primers: forward 5′CCTGCCCTTTACTTCGGGG3′, reverse 5′TGTTCCTCAAAATGCCGTGG3′ (512-bp product, DF-1 cells, and ESCs), and forward 5′TAGTGGTCGTATGTTTGC 3′, reverse 5′ TGATGAACCACCACCATG 3′ (809-bp product, embryos). The PCR products were treated with the T7EI enzyme.

TA clones (*n* = 30) of the PCR fragments were sequenced, and the efficiency of gene knockout was calculated as follows: gene knockout efficiency = bacterial mutants/total sequenced bacteria × 100%.

### Microinjection of the Cas9/gRNA plasmid into chicken embryos

The polyethylenimine (PEI)-encapsulated CRISPR/Cas9 vector was injected into chicken embryos, which were then sealed with paraffin and incubated at 38.5° for 4.5 d. Frozen sections were prepared and examined using an inverted fluorescence microscope. DNA was extracted for analysis using the T7EI assay.

### Data analysis

SPSS19.0 software was used to carry out the *t*-test analysis (*P* < 0.05 for significant differences, *P* < 0.01 for highly significant differences). EXCEL2003 was used to generate figures.

### Data availability

The authors state that all data necessary for confirming the conclusions presented in the article are represented fully within the article.

## Results

### CRISPR/Cas9 vector

The VK001-08 vector contains a T7 promoter to drive transcription of Cas9 mRNA (Figure 1A). To improve gRNA expression in avian cells, the human-derived U6 promoter was replaced with an avian-derived U6 promoter (Figure 1B). This vector efficiently expressed GFP in DF-1 cells (Figure 1C). We inserted the three gRNA sequences into the vector (Figure 1D).

### CRISPR/Cas9-mediated gene knockout in DF-1 cells

**Figure 2:**
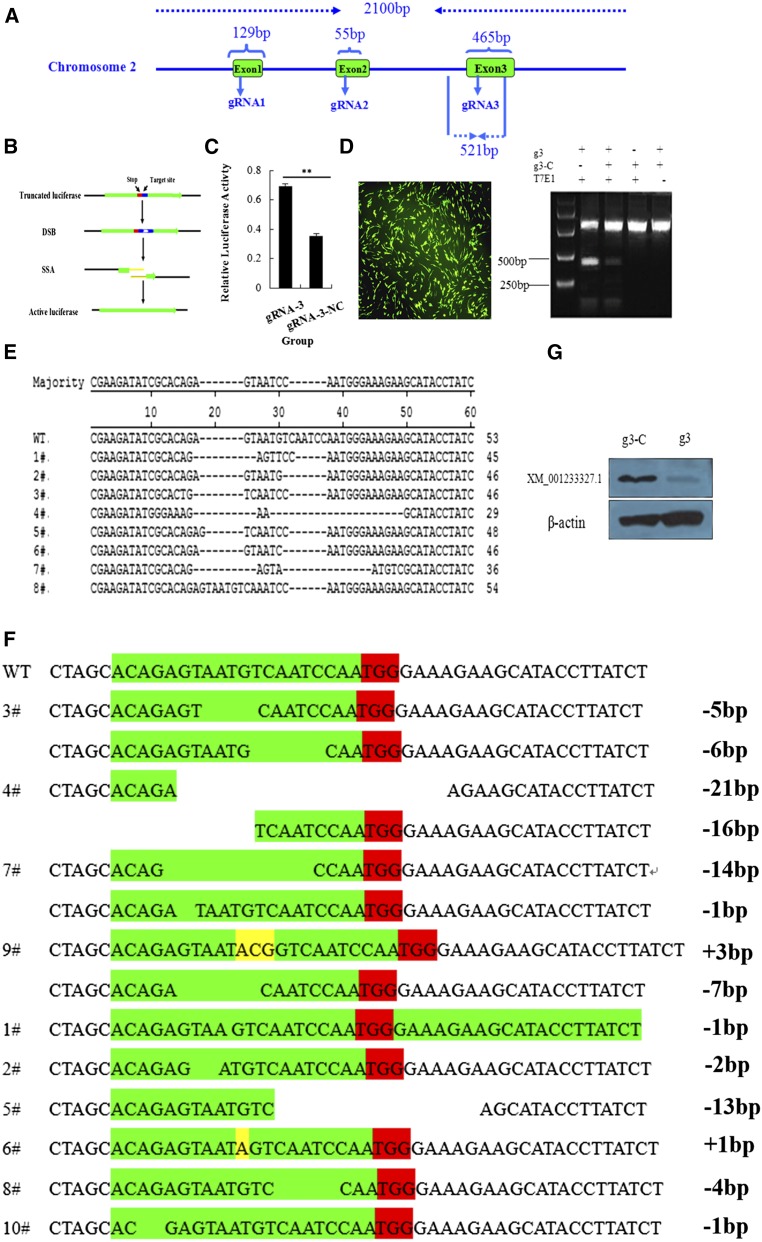
Cas9/gRNA-mediated gene deficiency in DF-1 cells. (A) Structure of the *C2EIP* gene and selection of the gRNA site. (B) Flowchart for the SSA activity assay. (C) Results of the SSA activity assay show high luciferase activity after transfection with the vector containing gRNA-3. (D) Right: results of the T7EI assay show a clear band at approximately 250 bp and gene knockout. Left: The effect of transfection in DF-1. (E) Alignment of TA clone sequences. (F) TA clone sequencing of monoclonal cells shows homozygous mutations in #3, #4, #7, and #9, and heterozygous mutations in the other cell lines. (G) Results of Western blot analysis show very low expression of C2EIP protein in monoclonal cells after transfection with the Cas9/gRNA plasmid.

The full-length target gene (*C2EIP*) was cloned, and three target sites were identified to construct the Cas9/gRNA vector (Figure 2A). We evaluated knockout efficiency using the SAA assay (Figure 2B), which showed the highest luciferase activity with the vector containing gRNA-3 (Figure 2C). We transfected DF-1 cells with the gRNA-3 vector, extracted genomic DNA 48 hr later, and cloned a 512-bp fragment containing the target site. Results of the T7EI assay showed cleavage products of the vector containing gRNA-3 (Figure 2D). TA clone sequencing showed a knockout efficiency of 27% (8/30) (Figure 2E). We then obtained 10 monoclonal cell lines using the limiting dilution method. Results of TA clone sequencing revealed homozygous mutations in four cell lines, and heterozygous mutations in six cell lines (Figure 2F). Homozygous mutations were associated with very low C2EIP protein expression (Figure 2G).

### CRISPR/Cas9-mediated gene deficiency in chicken ESCs

**Figure 3:**
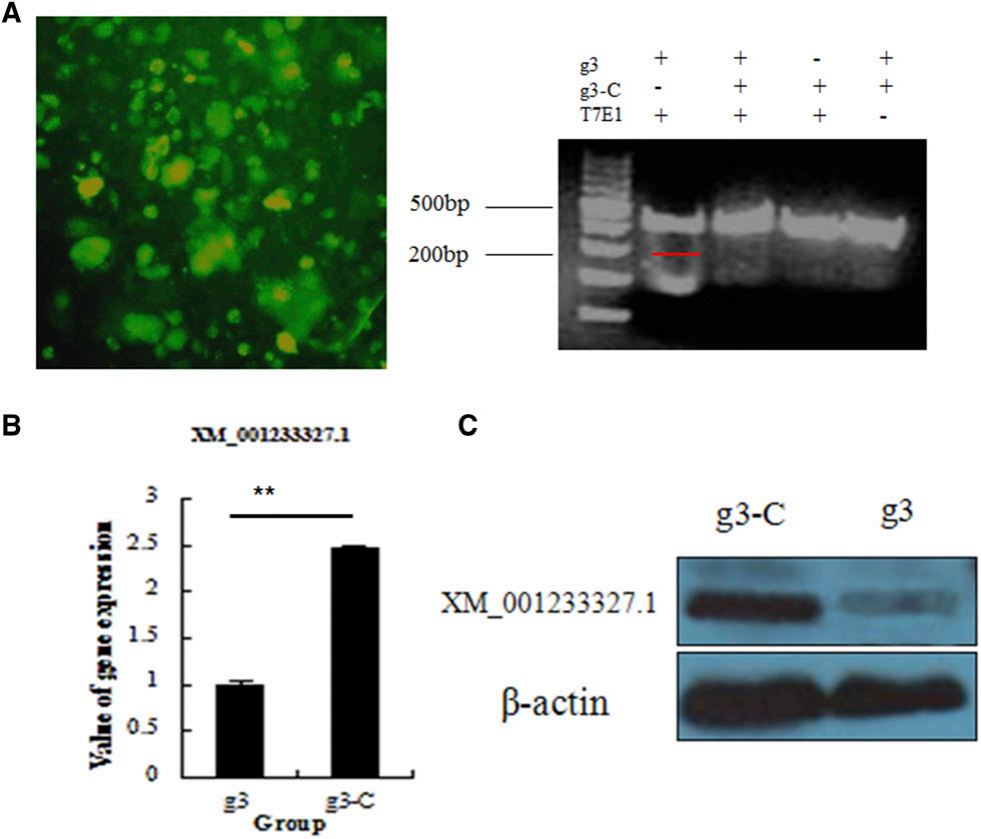
CRISPR/Cas9-mediated gene deficiency in chicken ESCs. (A) Effect of CRISPR/Cas9 plasmid transfection into chicken ESCs. Right: results of the T7EI assay indicate *C2EIP* gene knockout. (B) Results of qPCR and Western blot analysis show downregulated expression of the *C2EIP* gene and protein (** *P* < 0.01).

To determine whether CRISPR/Cas9 gene editing can knockout genes in stem cells of the domestic chicken, we tested the vector containing gRNA-3 in purified second-generation chicken ESCs (Figure 3A). At 48 hr after transfection of the vector, genomic DNA was extracted, and a fragment of approximately 512 bp containing the target site was cloned. Cleavage products were obtained after treating the cloned fragment with T7EI, and the knockdown efficiency was 27% (Figure 3B). Results of quantitative PCR (qPCR) and Western blot analysis showed decreased levels of C2EIP mRNA and protein (Figure 3, B and C).

### CRISPR/Cas9-mediated gene deficiency in chicken embryos

**Figure 4:**
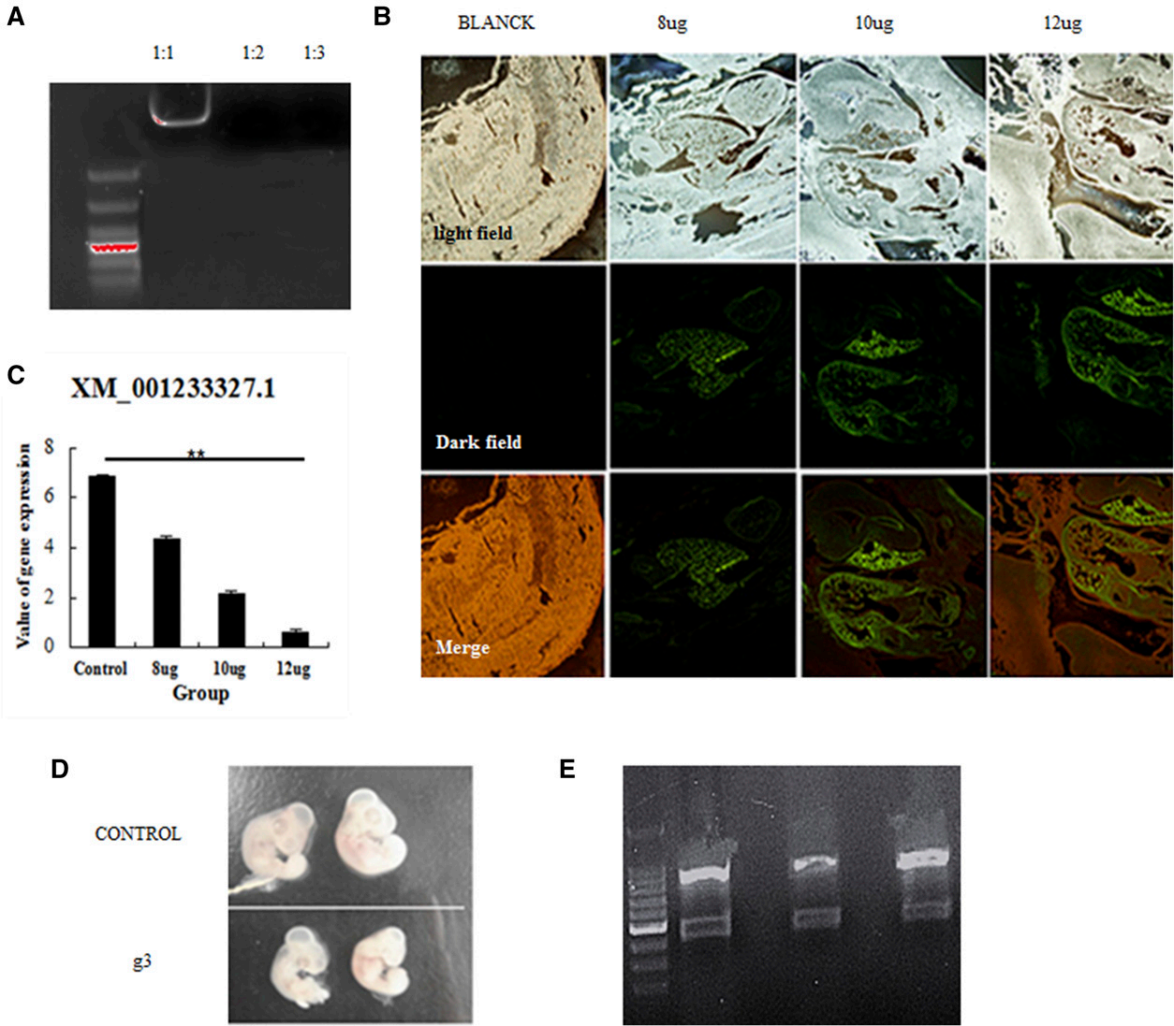
CRISPR/Cas9-mediated gene deficiency in chicken embryos. (A) PEI encapsulation of the CRISPR/Cas9 vector was evaluated by electrophoretic mobility shift assay. (B) Expression of the vector in chicken embryos, as assessed in frozen sections. (C) Downregulation of the *C2EIP* gene after microinjection with the CRISPR/Cas9 vector, as assessed by qPCR (** *P* < 0.01). (D) Comparison of microinjected and control chicken embryos. (E) Results of the T7EI assay showed cleavage products for three of the 20 chicken embryos.

To determine whether the CRISPR/Cas9 system can knockout genes in chicken embryos, the PEI-encapsulated vector containing gRNA-3 was injected into chicken embryos (Figure 4A). Analysis of embryo frozen sections showed that the plasmid was expressed, with the highest GFP expression observed in the heart (Figure 4B). Results of the T7EI assay showed the expected cleavage products, and qPCR results demonstrated lower expression of *C2EIP* in the transfected chicken embryos (Figure 4C and D). The efficiency of gene knockdown was 15% (3/20) (Figure 4E).

## Discussion

Successful gene knockout allows investigators to study gene function and identify redundant and epistatic genes. Investigators have attempted site-directed modification of target genes using natural DNA repair mechanisms; however, the efficiency of natural recombination is low and lacks repeatability. Simpler and more effective approaches to gene knockout/knock-in have been developed, including engineered endonuclease techniques. ZFN (Xiao *et al.* 2011) and TALEN (Boch and Bonas 2010; Bonas *et al.* 1989) are widely used tools, but the construct design and experimental procedures are complex. CRISPR/Cas9 is replacing ZFN and TALEN technologies because it is simpler and faster (Mussolino and Cathomen 2013).

Gene editing using the CRISPR/Cas9 system has been well developed, allowing the knockout of single or multiple genes simultaneously. CRISPR/Cas9 has been used to generate stable knockout cell lines (HEK293 cells, induced pluripotent stem cells) and knockout animals (mouse, rat, and zebrafish) using microinjection techniques. Sternberg *et al.* (2014) and Jinek *et al.* (2014) used this technique to generate DNA double-strand breaks, suggesting that the CRISPR/Cas9 system is suitable for gene editing in humans. Cho *et al.* (2014) demonstrated that increasing gRNA concentration could improve gene knockout efficiency in a cotransfection system, reporting an efficiency close to 33%. Sommer *et al.* (2014) modified the Cas9 system and used it for gene knockout in human HEK293 and K562 cells. Cong *et al.* (2013) knocked out the *EMX1* and *PVALB* genes in HEK293 cells and the *Th* gene in mouse Neuro 2A cells using the CRISPR/Cas9 system. Cong and Zhang (2015) and Mali *et al.* (2013) demonstrated higher knockout efficiency when the gRNA is structurally similar to the crRNA:tracrRNA complex. Mali *et al.* (2013) reported knockout efficiencies of 10–25% in HEK293 cells, 8–13% in K562 cells, and 2–49% in induced pluripotent stem cells. Yang *et al.* (2014) reported knockout efficiency close to 40%, targeting single, double, and multiple genes in mouse ESCs (*Tet1*, *Tet2*, *Tet3*, *Uty*, and *Sry*), as demonstrated by the restriction fragment length polymorphism assay, sequencing, and Southern blot analysis. Zhang *et al.* also modified multiple genes in mouse cells.

Although Zhang *et al.* reported that the CRISPR/Cas9 system was suitable for editing any gene, the latter study evaluated the technique only in mammalian cells. Recently, nice results from two studies using chicken cell lines have been published. In the studies conducted by Veron and coworkers, expression levels of somatic cells in chicken embryos were modified by electroporation of CRISPR gRNA plasmids directed against the PAX7 transcription factor (Nadège *et al.* 2015), Bai and coworkers edited the PPAR-γ, ATP synthase epsilon subunit (ATP5E), and ovalbumin (OVA) genes in chicken DF-1 cells using CRISPR strategies (Bai *et al.* 2016). However, all these studies show only that the CRISPR/Cas9 can knock out the gene in DF-1, but not in embryonic stem cells or embryos. For that reason, we evaluated the gene knock-down efficiency of this technique in somatic founder cells, nonfounder ESCs, and embryos of Suqin yellow chickens. The knock-down efficiency in our study (15–27%) was lower than that found in mammals and plants (40–80%), and was the first CRISPR/Cas9-based gene knock-down experiment in chicken embryonic stem cells and embryos. The technology and cell numbers are limiting factors for high gene knock-down efficiency. Nevertheless, our results support the application of CRISPR/Cas9 for gene editing in the domestic chicken, providing a new method for characterizing gene function in this species.
